# Chronic stress inhibits weight gain and decreases beta-hydroxybutyrate levels and glucose tolerance in female BALB/c fed a high-fat ketogenic diet

**DOI:** 10.1186/s40795-025-01129-8

**Published:** 2025-07-25

**Authors:** Ana L. Cantú-Ruiz, Diana Caballero-Hernández, Isaias Gutierrez-Leal, Ana C. Martínez-Torres, Ricardo Gomez-Flores, Patricia Tamez-Guerra, Reyes Tamez-Guerra, Cristina Rodríguez-Padilla

**Affiliations:** Universidad Autónoma de Nuevo León, Facultad de Ciencias Biológicas, Departamento de Microbiología e Inmunología, Laboratorio de Inmunología y Virología, San Nicolás de los Garza, Nuevo León, 66451 México

**Keywords:** Chronic stress, Ketogenic diet, Β-hydroxybutyrate, Obesity-resistance, Glucose tolerance, Sex-dependent

## Abstract

**Introduction:**

Known for its neuroprotective properties, the ketogenic diet (KD) recently has been shown to prevent weight loss induced by chronic stress in rats, although the mechanisms remain to be elucidated. The obesity-resistant BALB/c mouse is susceptible to chronic stress–induced weight loss, providing a useful model to study the interactions between diet and stress.

**Objective:**

This study aimed to evaluate the potential of a ketogenic diet to prevent chronic stress-induced weight loss in the obesity-resistant BALB/c mouse strain.

**Method:**

BALB/c mice of both sexes, were divided into groups: (1) standard chow, (2) KD, (3) standard chow + stress, and (4) KD + stress. The stress groups were subjected to a restraint stress protocol for 23 d, 4 h a day. Morphometric changes, glucose tolerance, plasmatic corticosterone levels, and circulating ketone bodies were evaluated.

**Results:**

Levels of β-hydroxybutyrate increased in the KD group in both sexes. However, under stress, the increase in ketone bodies was lower in female mice. Compared with standard chow-fed groups, females on a KD gained significant body weight, an effect lost in females under stress, with decreasing fat tissue deposits. In male mice, although no changes in body weight were observed in the KD group, the mass of adipose tissue depots increased and remained unchanged under stress. Under chronic stress both standard chow and KD-fed mice lost weight. Under KD, female and male BALB/c mice exhibited decreased water and food intake, as well as reduced glucose tolerance, under resting and chronic stress conditions.

**Conclusions:**

There is an interplay between chronic stress and ketogenic metabolism in BALB/c mice. In female mice, chronic stress interferes with ketogenesis, lowering beta-hydroxybutyrate levels and preventing weight gain whereas the KD inhibits chronic stress-induced glucose tolerance, this in a sex-dependent manner.

**Supplementary Information:**

The online version contains supplementary material available at 10.1186/s40795-025-01129-8.

## Introduction

Chronic stress is considered a negative emotional experience with the potential to disrupt the body’s homeostasis. The dysregulation of this homeostasis due to stressors plays a role in the development of various pathologies, such as infections, metabolic imbalances, neurodegenerative diseases, and cancer [1]. It is known that stress activates the nervous system, triggering the sympathetic-adreno-medullary (SAM) pathway and the hypothalamic-pituitary-adrenal (HPA) axis, releasing hormones such as catecholamines and glucocorticoids, which chronically impact individuals’ health [2]. Recently, due to the neuroprotective properties attributed to the consumption of a ketogenic diet (KD) [3–5], as well as the ability of ketone bodies to cross the blood-brain barrier to supply energy to the brain, the modulatory role of this diet under chronic conditions such as stress has been questioned.

The KD, widely used as a treatment for neurodegenerative diseases such as epilepsy, is considered a high-fat diet with an appropriate protein content and restricted carbohydrates, forcing the body to alter its metabolism by maintaining a ketogenic state due to high levels of ketone bodies in the blood [6, 7]. It has been reported that the administration of a KD has protective effects on mood disorders, reduction of glucose levels, improved insulin sensitivity, anti-inflammatory properties, and body weight loss [8–11]. The effects of KD, such as body weight loss, are attributed to metabolic adjustments involving adrenergic receptors [12]. Similarly, a direct relationship between ketone bodies and the regulation of the nervous system, controlling the body’s energy expenditure for the maintenance of metabolic homeostasis has been reported, placing the nervous system as a mediator of the effects of the KD on body weight [13, 14].

Due to the neuroprotective properties attributed to the consumption of the KD [3–5], the modulating role that this diet might have in chronic conditions such as stress has been investigated. In this regard, it has been demonstrated that a ketogenic diet has beneficial effects in individuals who consume it regularly, improving mood, mental well-being, and cognitive function while reducing stress, depression, and anxiety compared to those following other types of diets [15].

Limited studies show that consuming a ketogenic diet (KD) prevents the effects of chronic stress, particularly body weight loss [16]. In addition, it has been reported that under acute stress conditions, circulating ketone bodies increase [17, 18], thereby attenuating the stress-induced rise in inflammatory cytokines such as IL-1β and TNFα [19, 20]. Despite the widespread use of the KD, the regulatory mechanism of its effects under chronic stress conditions has not yet been elucidated.

Recently, a study evaluating sex- and age-related differences in mice on a ketogenic diet, observed glucose intolerance in males and increased adiposity in females [21]. In the last decade, increasing importance has been given to conducting research on both sexes. In the past, many studies were performed primarily on males to avoid the variability introduced by the estrous cycle and female sex hormones [22]. To ensure that these findings are useful for understanding the effects of KD consumption in humans, it is essential to investigate the impact of the ketogenic diet in both sexes.

The present study aims to evaluate the potential of a KD to prevent chronic stress-induced weight loss in the obesity-resistant BALB/c mouse, circulating ketone bodies, weight gain, adipose tissue deposits, feeding behavior, and glucose tolerance were assessed. Our findings shed light on the interaction between chronic stress and ketogenic metabolism in a widely used lab mouse strain, as well as their differential effect by sex.

## Methods

### Animals

We used 12- to 16-week-old BALB/c mice of both sexes obtained from the bioterium of the Facultad de Ciencias Biológicas at Universidad Autónoma de Nuevo León (UANL), México. Mice were provided with food and water *ad libitum* and housed in controlled microenvironmental conditions of 12 h:12 h light: dark cycles with controlled temperature ranging between 22 and 25 °C and 45% relative humidity.

All experimental procedures in animals were approved by the Research Ethics and Animal Welfare Committee of Facultad de Ciencias Biológicas, UANL (CEIBA-2021-002).

### Experimental design

To reduce stress induced by handling, animals were daily handled for five minutes by the same operator. They were randomly divided into the following groups: males; standard chow (*n* = 5), standard chow + stress (*n* = 5), KD (*n* = 5), and KD + stress (*n* = 4) and females; standard chow (*n* = 10), standard chow + stress (*n* = 8), KD (*n* = 5), and KD + stress (*n* = 5). Mice were subjected to daily restraint stress for four hours during 23 d. For this procedure, mice were individually placed in well-ventilated 50 mL conical tubes [23]. Standard chow consisted of 13% fat, 28% protein, and 57% carbohydrate (LabDiet 5001, 2.86 kcal g, LabDiet, St. Louis, MO, USA), whereas the KD consisted of 80% fat, 15% protein, and 5% carbohydrates (Research Diets D06040601, 6.1 kcal g; Research Diets Inc., New Brunswick, NJ, USA) [24, 25]. Food and water intake were assessed through the protocol. Mouse body weight was measured every two days, using a digital scale.

### Glucose tolerance test

On day 23 of the protocol, we assessed glucose tolerance [26]. Mice were fasted for six hours, blood was collected from the tip of the tail and baseline blood glucose was measured using a glucometer (Trividia Health Inc., Fort Lauderdale, Florida, USA) (time 0). Next, a 100 mg/mL glucose solution (ICN Biomedicals, Aurora, OH, USA) in sterile saline solution, was administered by intraperitoneal injection. Blood glucose was measured at 15, 30, 60, and 120 min post-glucose injection. The area under the curve (AUC) was calculated using the glucose time-course data.

### β-hydroxybutyrate plasma levels

Before euthanizing the animals, changes in blood β-hydroxybutyrate levels (BHB), the primary circulating ketone bodies, were determined by measuring the blood ketone body concentration immediately after blood collection, using the KetoSens™ test strips (i-SENS, Seoul, Korea), following manufacturer´s instructions.

### Tissue sampling

At the end of the experiment, mice were anesthetized with a dose of 100 mg/Kg pentobarbital (Aranda, Mexico) and terminal cardiac puncture was conducted to obtain blood samples, after which animals were euthanized by cervical dislocation. The interscapular, inguinal, and visceral adipose tissue deposits were removed, weighed on an analytical scale, and stored at -80 ºC until analysis. Body mass index (BMI) was calculated by dividing the body weight by the square of the nose-anus length at the end of the experiment.

### Plasma corticosterone

To determine HPA activation an enzyme-linked immunosorbent assay (ELISA, Parameter Corticosterone Assay KGE009, R&D Systems) was conducted to quantify plasmatic corticosterone levels, following the manufacturer’s instructions. All samples were analyzed in duplicate to ensure accuracy.

### Statistical analysis

Data are expressed as mean ± SE. Statistical analyses were performed using GraphPad Prism 8.0 software (San Diego, CA, USA). Water and food intake, BMI, ketone body levels, AUC, corticosterone levels, and percentage of adipose tissue depots were first analyzed by one-way ANOVA, followed by planned multiple comparisons. If significant results were obtained, a Bonferroni post-hoc test was performed. Body weight and glucose tolerance test were assessed by two-way ANOVA, if significant results were obtained, a Tukey post-hoc test was performed. *P* values *≤* 0.05 were considered statistically significant.

## Results

### Plasma β - hydroxybutyrate levels

**Fig. 1:**
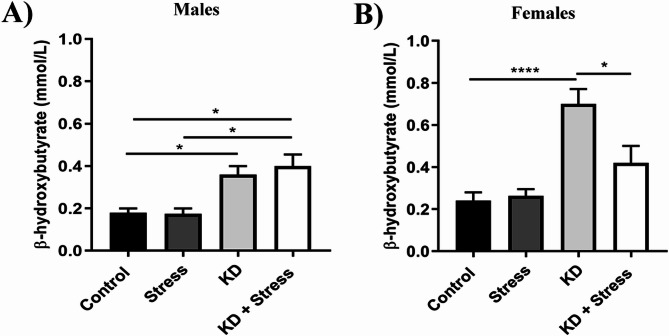
Effect of the KD on BHB levels in BALB/c mice under conditions of rest and chronic stress. Mice consumed a standard diet or a KD for 23 d. On day 23 of the protocol, we evaluated blood ketone bodies. Levels of BHB in blood in (**A**) male and (**B**) female mice of the BALB/c strain at day 23 under a KD and chronic stress. In BALB/c mice on a KD, blood ketone body levels increased, under stress or resting conditions. However, in females, ketone body levels were lower under chronic stress conditions; *n* = 4–10 mice/ group. Data represent the mean ± standard error. *P* values ≤ 0.05 were considered statistically significant

β-hydroxybutyrate levels were measured to assess the activation of ketogenesis when consuming a ketogenic diet (males, *n* = 4–5 mice / group; females, *n* = 5–10 mice / group). In males consuming a KD, an increase in BHB levels was observed both under resting (0.3 mmol/L, *p* = 0.0474) and chronic stress (0.4 mmol/L, *p* = 0.0269) conditions (Fig. 1A). In contrast, although females consuming KD also showed an increase in BHB levels (0.7 mmol/L, *p* = 0.0001); in those subjected to chronic stress, the increase was significantly reduced (0.42 mmol/L, *p* = 0.0149) (Fig. 1B).

### Body weight

To assess whether the consumption of a KD induces changes in body weight in BALB/c mice under conditions of rest or chronic stress, we monitored body weight changes through the protocol (males, *n* = 4–5 mice / group; females, *n* = 5–10 mice / group).

**Fig. 2:**
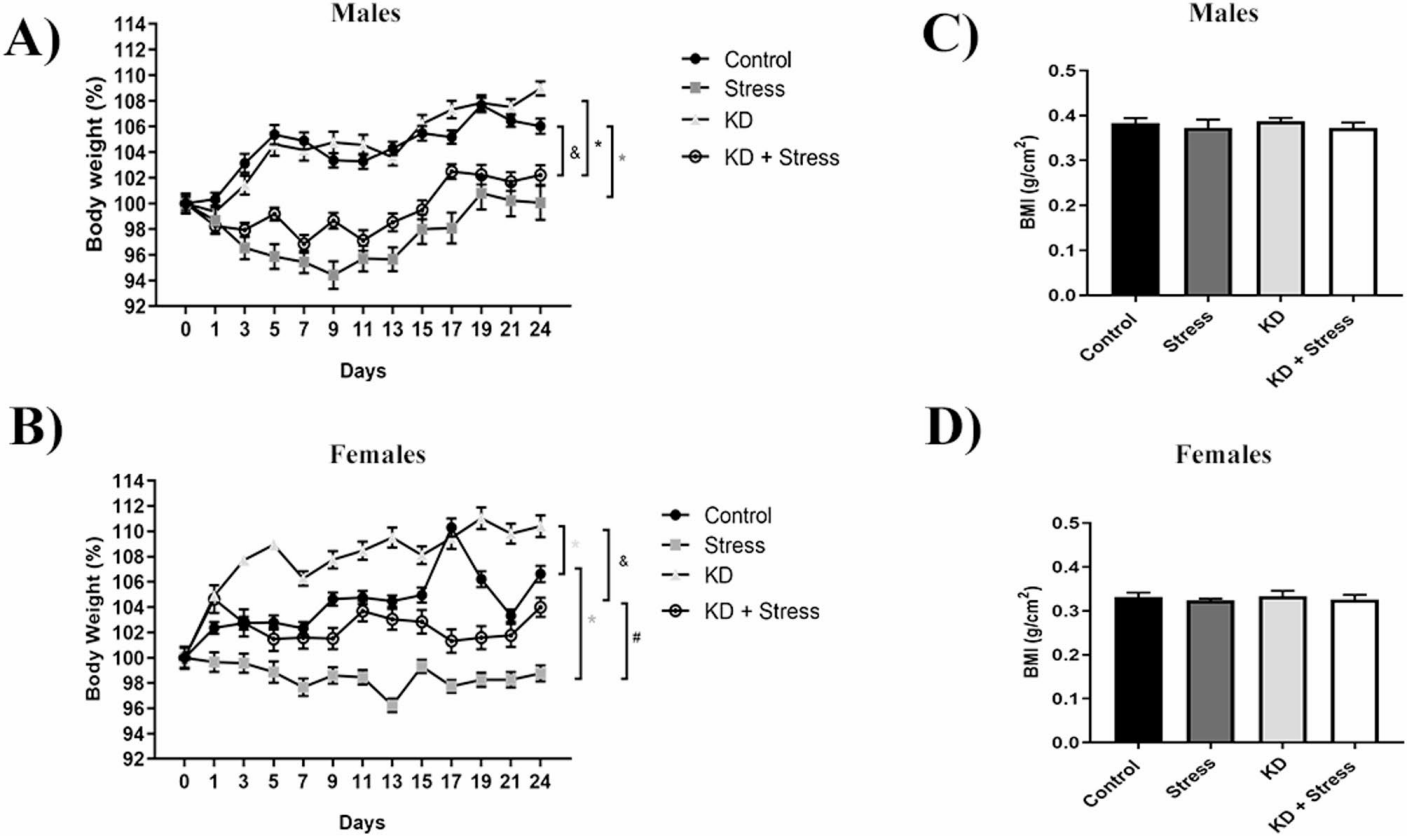
Evaluation of body weight gain during 23 d of the protocol. Mice were divided into four groups and subjected to a chronic restraint stress protocol, a chow diet, and a KD. Results were reported as a percentage of body weight. In males on a KD, a decrease in body weight was observed under chronic stress, when consuming a standard and a KD. In females, mice that consumed a KD increased their body weight, whereas in chronic stress they lost weight. No alterations in BMI were observed in any of the conditions evaluated. Body weight gain in (**A**) male and (**B**) female mice of the BALB/c strain, during 23 d of chronic stress and consumption of a KD. IBM in (**C**) male and (**D**) female BALB/c mice during 23 d of chronic stress and consumption of a KD; *n* = 4–10 mice/ group. Data represent the mean ± standard error. *P* values ≤ 0.05 were considered statistically significant

We found that the consumption of a KD does not alter the body weight of male mice. However, when subjected to stress, male mice lost 6% of their body weight by day 23 of the protocol, regardless of whether they consumed a standard (*p* = 0.0001) or a KD (*p* = 0.0001) diet (Fig. 2A). However, we did not observe changes in BMI (Fig. 2C).

Female mice consuming a KD gained 4% more body weight (*p* = 0.0008), this weight gain was not observed under stress conditions, where the mice lost 6% of their body weight (*p* = 0.0364). Female mice fed a standard diet and chronically stressed lost 7% of their body weight, as compared with resting mice (*p* = 0.0001) (Fig. 2B). No changes in BMI were observed under KD or stress conditions (Fig. 2D).

### Body fat mass

Animals were euthanized and tissues excised, the interscapular, inguinal and visceral adipose tissue deposits of all experimental groups were weighed.

**Fig. 3:**
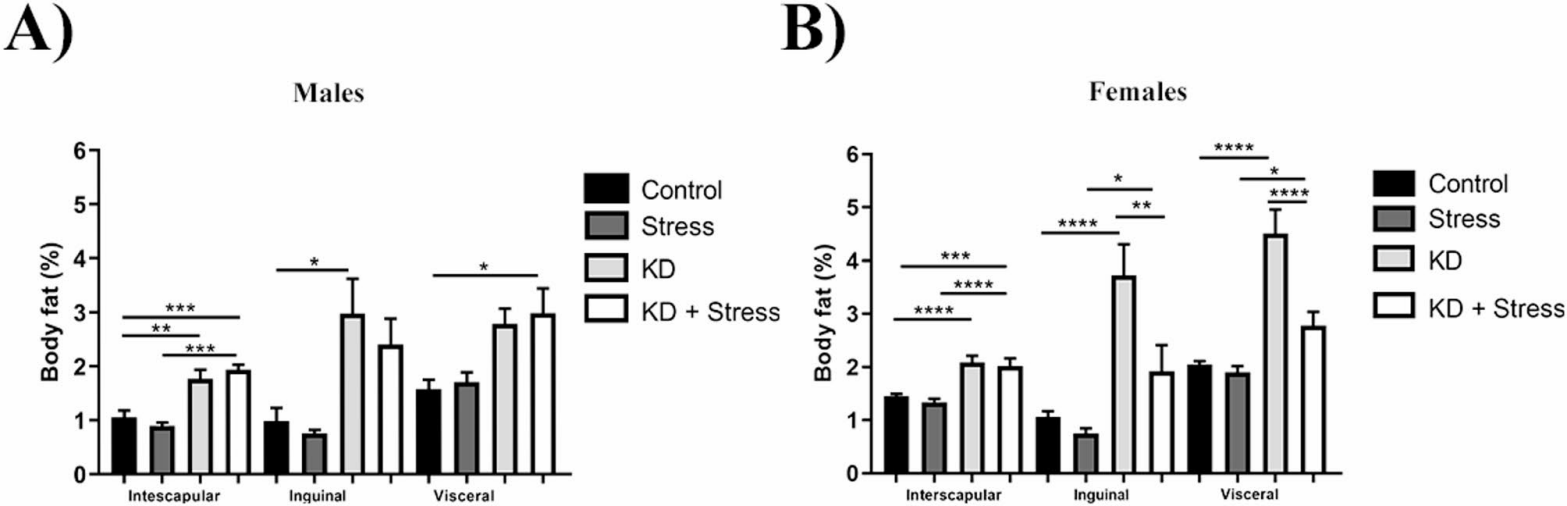
Evaluation of body fat mass changes at day 23 of restraint stress, KD, or their combination. In mice that consumed a KD, an increase in interscapular, inguinal, and visceral tissue deposits was observed in both sexes. In stressed mice, only females with KD showed a decrease in inguinal and visceral tissue deposits. Body fat mass in (**A**) male and (**B**) female BALB/c mice at day 23 of chronic stress and consumption of a KD; *n* = 4–10 mice/ group. Data represent the mean ± standard error. *P* values ≤ 0.05 were considered statistically significant

In male mice (*n* = 4–5 / group) consuming a KD, interscapular fat increased from 1.05 to 1.77% (*p* = 0.0040) and inguinal fat increased from 0.98 to 2.97% (*p* = 0.0285). In addition, male mice on the KD subjected to chronic stress showed an increase in interscapular fat from 1.05 to 1.9%, as compared to resting (*p* = 0.0006) or stressed mice on a standard diet (*p* = 0.0002). Similarly, male mice that consumed KD and were subjected to chronic stress exhibited an increase in visceral fat from 1.57 to 2.98% (*p* = 0.0237) (Fig. 3A).

In female mice (*n* = 5–10 / group) consuming a KD, an increase in interscapular fat from 1.4 to 2.08% (*p* = 0.0001), inguinal fat from 0.98 to 2.97% (*p* = 0.0285), and visceral fat from 2.04 to 4.5% (*p* = 0.0001) was observed. However, when mice on KD were subjected to chronic stress, both inguinal (1.91%) and visceral (2.7%) fat significantly (*p* = 0.0025) decreased (Fig. 3B).

### Feeding and drinking behavior

To assess whether food and water consumption contributed to the increase in body weight and fat in the mice, we evaluated water, food and energy intake in our experimental groups (males, *n* = 4–5 mice / group; females, *n* = 5–10 mice / group).

**Fig. 4:**
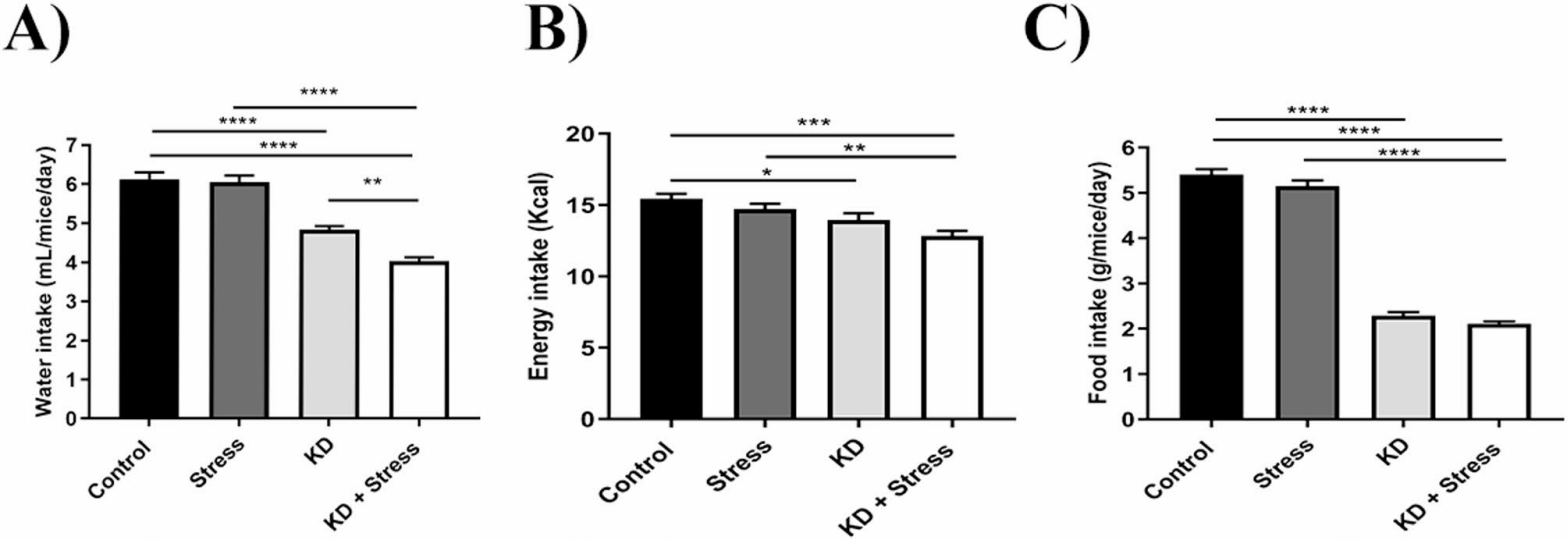
Effect of a KD on eating behavior in male mice under chronic stress. In mice under a KD, a decrease in water, energy, and food intake was observed, as compared with the control group. However, in mice with KD under stress, a decrease in water consumption was observed. Average intake of (**A**) water and (**B**) energy intake, and (**C**) food of male BALB/c mice under a KD and chronic stress; *n* = 4–10 mice/ group. Data represent the mean ± standard error. *P* values ≤ 0.05 were considered statistically significant

Males on a KD consumed 50% less water than mice on a standard diet (*p* = 0.0001). This decrease in water consumption was more pronounced in mice that were on a KD and subjected to chronic stress (4.02 mL, *p* = 0.0018) (Fig. 4A). When evaluating energy intake, male mice had a lower energy intake (12.8 kcal/day) under a KD both at resting (*p* = 0.0494) and under stress (*p* = 0.0059) conditions (Fig. 4B). Food intake decreased in male mice on a KD (Fig. 4C), regardless of whether they were at resting (1.65 g/day, *p* = 0.0001) or under stress (1.84 g/day, *p* = 0.0001), compared to mice on a standard diet (5.40 g/day).

**Fig. 5:**
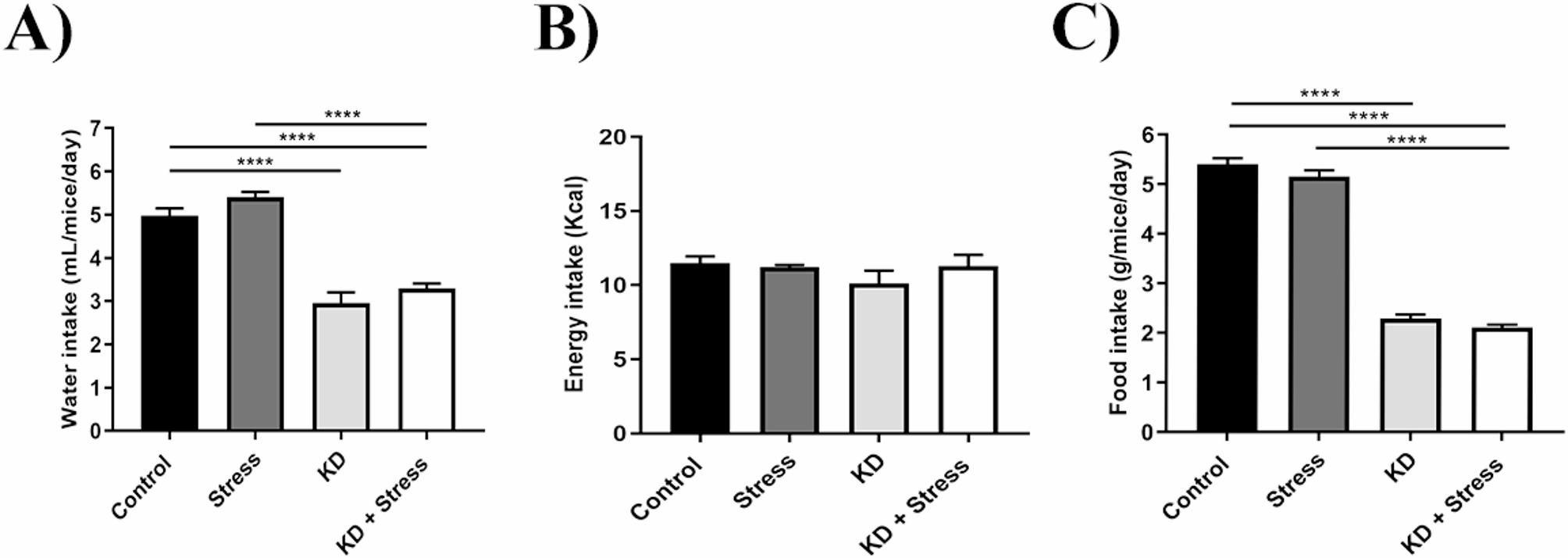
Effect of a KD on eating behavior in female mice under chronic stress. In mice under a KD, a decrease in food and water consumption was observed, as compared with the control group. Average intake of (**A**) water and (**B**) energy, and (**C**) food of female BALB/c mice under a KD and chronic stress; *n* = 4–10 mice/ group. Data represent the mean ± standard error. *P* values ≤ 0.05 were considered statistically significant.

Similarly, female mice water intake decreased (2.96 g/day, *p* = 0.0001) under KD (Fig. 5A). We did not observe significant differences in energy intake (Fig. 5B). Food intake decreased under a KD (Fig. 5C), both at rest (1.65 g/day, *p* = 0.0001) and under chronic stress (1.84 g/day, *p* = 0.0001), as compared with those on a standard diet (4.01 g/day).

### Glucose tolerance

**Fig. 6:**
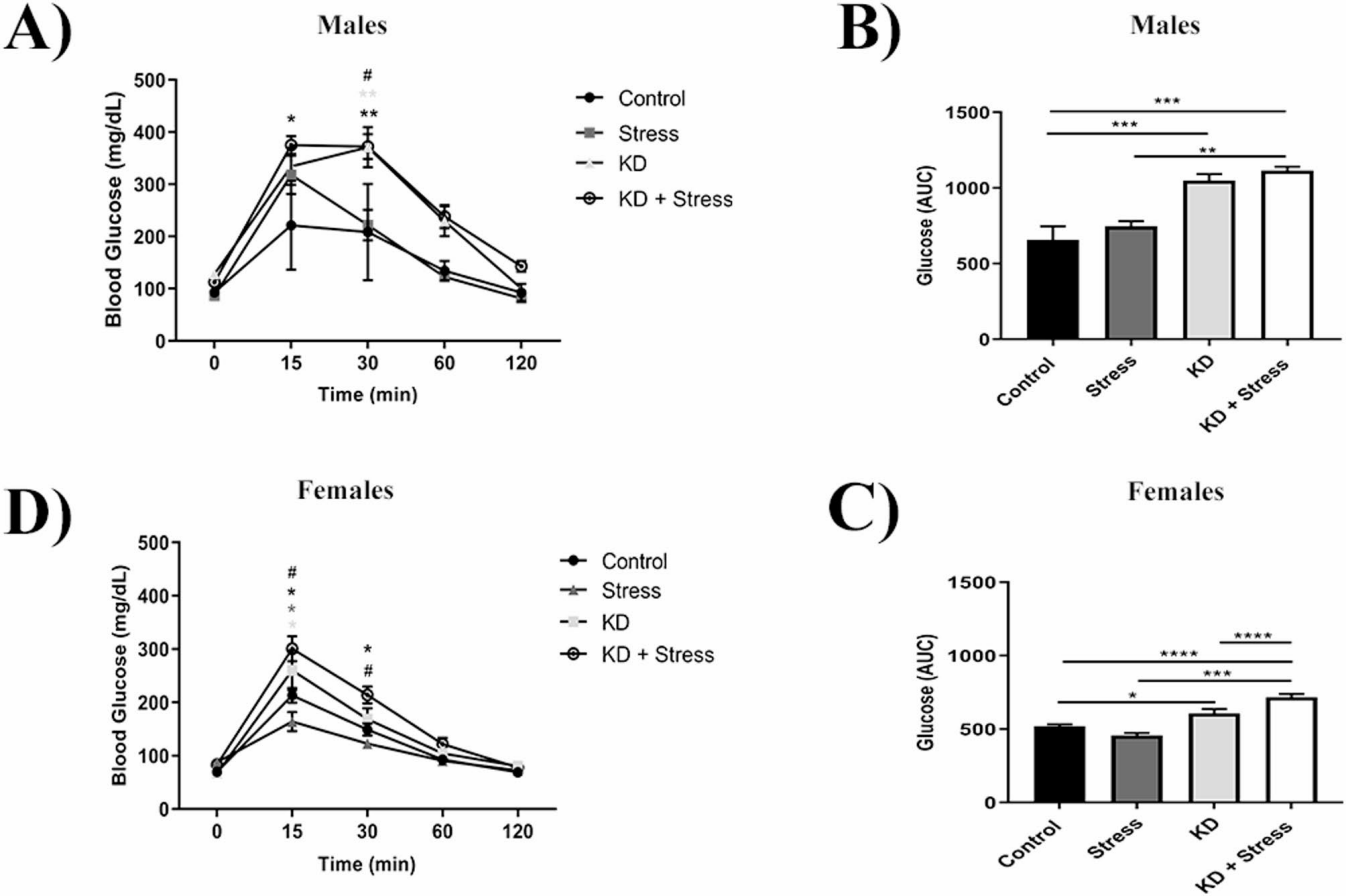
Effect of a KD, chronic stress, and their combination in BALB/c mice on day 23 of the protocol. Mice were fasted for six hours, after which blood was collected from the tip of the tail and a determination of basal blood glucose was performed using a glucometer. After intraperitoneal glucose injection, blood glucose levels were measured at 15, 30, 60, and 120 min. Mice that were under a KD had lower glucose tolerance in both sexes. Glucose tolerance test in (**A**) male and **C**) female BALB/c mice on day 23 of the protocol. Area under the curve in (**B**) male and **D**) female mice under a KD and chronic stress due to movement restriction; *n* = 4–10 mice/ group. Data represent the mean ± standard error. *P* values ≤ 0.05 were considered statistically significant.

In male mice on a ketogenic diet, lower glucose tolerance was observed both at resting conditions (minute 30; *p* = 0.0082) and chronic stress (minute 15; *p* = 0.0138 and minute 30; *p* = 0.0273). This was consistent with the AUC, for which an increase in area was observed in mice consuming a ketogenic diet both at resting (*p* = 0.0007) and chronic stress conditions (*p* = 0.0002) (Fig. 6A and B) (*n* = 4–5 mice / group).

In female mice on a KD, lower glucose tolerance was observed at resting (minute 15; *p* = 0.0421) and under chronic stress conditions (minute 15; *p* = 0.0001 min 30; *p* = 0.0019). In contrast, mice under chronic stress on a standard diet showed improved glucose tolerance (minute 15; *p* = 0.0087). When evaluating the AUC, an increase was observed in the KD group (*p* = 0.0187) and in the KD group under stress (*p* = 0.0001) (Fig. 6C and D) (*n* = 5–10 mice / group).

### Plasmatic corticosterone levels

**Fig. 7:**
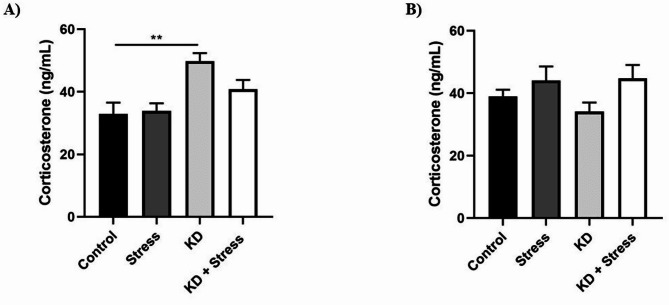
Evaluation of plasma corticosterone levels in BALB/c mice under resting conditions, restraint stress, KD, or their combination. Males that consumed KD for 23 days showed an increase in plasma corticosterone levels, but not females. Corticosterone levels in BALB/c mice: (**A**) males and (**B**) females under mobility restriction, KD, or their combination; *n* = 4–10 mice / group. Data represent the mean ± standard error. *P* values ≤ 0.05 were considered statistically significant.

In male mice (*n* = 4–5 mice/group) that consumed KD, plasma corticosterone levels significantly increased from 32.88 ng/mL to 49.73 ng/mL compared to the control group (*p* = 0.0086) (Fig. 7A). In contrast, no significant changes were observed among female groups, or by stress induction (Fig. 7B).

## Discussion

In this study, we evaluated the effect of a ketogenic diet in chronically stressed obesity-resistant BALB/c mice. Our results showed that KD and chronic stress independently influenced body weight and glucose tolerance in female BALB/c and their interaction alters the direction and magnitude of these responses.

To confirm that the KD used in this study induced nutritional ketosis; we evaluated blood levels of β-hydroxybutyrate, a ketone body considered responsible for anti-inflammatory and neuroprotective effects observed against chronic degenerative diseases [20]. Circulating ketone bodies are produced under different conditions in the body, including starvation, fasting, prolonged exercise or dietary regimens such as the KD [6, 7]. In our study, the animals fed a KD developed higher levels of ketone bodies, compared with the groups fed a standard diet, which agrees with previous reports where the effects of this diet were studied [25, 27, 28]. Regarding the impact of stress on ketone bodies levels, it has been observed in various reports that during acute stress, plasma levels of BHB increase [17, 29, 30]. Others have reported that in C57BL/6 mice subjected to acute social defeat stress (SDS), BHB levels increased [18], whereas a chronic stress protocol did not significantly affect plasma BHB levels, consistent with our results. We observed no changes in BHB levels in animals under chronic stress and a standard diet, compared with groups on resting conditions.

However, for animals under KD, although increasing levels were observed in both sexes under chronic stress, levels of BHB were lower in females, indicating that chronic stress suppresses the increase in levels of circulating ketone bodies in a sex-dependent manner in BALB/c mice, in contrast with others findings [16].

These sex-specific effects could be attributed to the estrous cycle in females, variations in female sex hormones, age, strain, or even the study model. Sex differences have been observed in plasma ketone body levels, with women exhibiting higher fasting ketone body levels and showing sex-dependent responses to metabolic challenges [31, 32]. However, the exact mechanism underlying these differences remains unclear. Additionally, it has been reported that males consuming a ketogenic diet (KD) are more susceptible to metabolic changes, such as increased insulin resistance and glucose intolerance, whereas females are more susceptible to morphometric changes, such as greater adiposity [21]. In a study of C57Bl/6J mice consuming a KD, females were more susceptible to the adverse metabolic changes observed [33].

Numerous reports have demonstrated the effects of consuming a KD on body weight, most of which report the loss of body weight and a decrease in body fat [12, 13]. This contrasts with the findings of our study, where we observed that females on a KD gained body weight and body fat, which was lost when animals were subjected to chronic stress, confirming the potential of the KD to reduce the effects of chronic stress on body weight. Others have observed that in Long-Evans rats subjected to unpredictable chronic stress for 21 days, a KD protected females from stress-mediated weight loss [16].

On the other hand, no changes in body weight were observed in male BALB/c mice fed with KD, whereas the groups under chronic stress lost weight regardless of the type of diet. Furthermore, we showed that body fat in male BALB/c mice increased when KD was administered, regardless of conditions, which may indicate that adipose depots are differentially affected by a KD. These sex-based differences might be related to the presence of sex hormones and their distinct roles in the mouse, with the presence of estrogen and the hormonal cycle [34]. For this study, the estrous cycle of the females was not evaluated to avoid any other stressors that may influence the results.

We assessed the intake of food and water, as behavior that may explain the weight gain. In both sexes, we found that when mice consumed KD, water and food daily intake decreased to half of what they consume when on standard chow. In this regard, we calculated energy intake, finding a decrease only in males, suggesting that food consumption is not related to body weight gain. It has been reported that KD induces satiety faster than standard chow, which agrees with our results. Therefore, it would be of interest to evaluate alterations in hormones such as leptin or ghrelin, as well as proteins involved in the regulation of metabolism in our future research. FGF21, for instance, has been directly correlated with metabolic regulation and thermogenesis, being one of the main regulatory proteins involved in the consumption of the KD, and one of the main candidates for its regulation [35, 36]. In addition, it has been shown that FGF21 is related to dietary intake [37]. The KD used in this study is primarily composed of soybean oil and lard, with both saturated and unsaturated fats, as well as a 15% protein content. Proteins may provide an energy source through gluconeogenesis, which may interfere with biological effects such as weight loss [38]. When consuming a low-carbohydrate diet, metabolism is altered because there is no glucose available as the primary source of energy. As a result, the body is forced to seek an alternative energy source through ketogenesis, leading to lower glucose levels and an improvement in insulin levels.

In our study, under this dietary regimen, mice that consumed the KD showed reduced glucose tolerance or elevated levels of glucose, which is conflicting with other authors’ reports, using the same mouse strain, the same percentage of macronutrients, or different diet administration protocols [36, 39]. On the other hand, some authors have reported comparable results to those found in our study, when the same diet was used [24, 40]. It has been demonstrated that the macronutrient composition of ketogenic diets can influence the observed effects, primarily those related to the composition of saturated fats in the diet [41, 42]. This also maintains a state of nutritional ketosis, which is consistent with our results.

When evaluating plasma corticosterone levels, no alterations were observed in the stressed groups, both in males and females. Although this contrasts with findings from various authors reporting increased serum corticosterone levels in response to different stressors [43, 44], it aligns with previous findings in the same model used in this study [23]. This contrast may be attributed to the habituation of mice to the stress paradigm. In this regard, it has been observed that 21 days of restraint stress led to habituation in mice, reflected in the absence of changes in corticosterone levels [45].

On the other hand, along with the increase in corticosterone, a decrease in body weight is observed throughout the stress paradigm, both parameters being indicative that the subjects are under stress [45, 46]. Although no changes in corticosterone levels were detected in our study, a significant loss of body weight was observed. The response to a stressor is influenced by multiple factors, including weight, age, strain, genetics, type of stimulus, and its duration, all of which play a crucial role in an individual’s adaptability to stress and its potential consequences [47].

When evaluating corticosterone levels in KD-fed mice, we observed an increase in plasma levels in male mice, whereas no changes were observed in females. The activity of the HPA axis has been linked to an individual’s nutritional status, particularly in the context of KD, through alterations in the production of metabolic hormones such as cortisol. In this regard, an increase in serum cortisol levels has been reported in Sprague-Dawley rats after two weeks on a KD [48]. Similarly, in male C57BL/6J mice and Long-Evans rats that consumed KD for three weeks, an increase in plasma corticosterone levels was observed, consistent with our findings [49]. This suggests that nutritional ketosis, induced by KD consumption, activates the HPA axis in a sex-dependent manner.

## Conclusions

In summary, chronic stress attenuates the effects of a KD in weight gain and BHB levels in female BALB/c mice. In contrast, the KD inhibits the increase in glucose tolerance observed in female BALB/c under chronic stress. These results suggest an interplay between chronic stress and a high-fat KD that modifies their effects in a sex-dependent manner. Additional research is necessary to investigate this interplay, as well as to assess the importance of sex hormones in the findings of this study.

## Electronic supplementary material

Below is the link to the electronic supplementary material.

Supplementary Material 1

## Data Availability

Data is available as supplementary material.

## Abbreviations

AUC: Area under curve
BHB: Beta-hydroxybutyrate
BMI: Body mass index
KD: Ketogenic diet
